# Supplementary material for: Homologous Recombination Repair Gene Alterations Are Associated with Tumor Mutational Burden and Survival of Immunotherapy
Source: Cancers (Basel). 2023 Nov 27;15(23):5608. doi: 10.3390/cancers15235608 (PMC10705153; doi:10.3390/cancers15235608)
Supplement: Supplementary file 1 [file cancers-15-05608-s001.zip › cancers-2736788-supplementary.pdf]

Supplementary data

Supplementary Table S1. List of genes analyzed with FoundationOne® CDx

A. gene list: the entire coding sequence was examined for the detection of base substitutions, insertion/deletions, and copy number alterations.

ABL1	*ACVR1B*	*AKT1*	*AKT2*	*AKT3*	*ALK*	*ALOX12B*	*AMER1*	*APC*	*AR*
ARAF	*ARFRP1*	*ARID1A*	*ASXL1*	*ATM*	*ATR*	*ATRX*	*AURKA*	*AURKB*	*AXIN1*
AXL	*BAP1*	*BARD1*	*BCL2*	*BCL2L1*	*BCL2L2*	*BCL6*	*BCOR*	*BCORL1*	*BRAF*
BRCA1	*BRCA2*	*BRD4*	*BRIP1*	*BTG1*	*BTG2*	*BTK*	*C11orf30*	*CALR*	*CARD11*
CASP8	*CBFB*	*CBL*	*CCND1*	*CCND2*	*CCND3*	*CCNE1*	*CD22*	*CD274*	*CD70*
CD79A	*CD79B*	*CDC73*	*CDH1*	*CDK12*	*CDK4*	*CDK6*	*CDK8*	*CDKN1A*	*CDKN1B*
CDKN2A	*CDKN2B*	*CDKN2C*	*CEBPA*	*CHEK1*	*CHEK2*	*CIC*	*CREBBP*	*CRKL*	*CSF1R*
CSF3R	*CTCF*	*CTNNA1*	*CTNNB1*	*CUL3*	*CUL4A*	*CXCR4*	*CYP17A1*	*DAXX*	*DDR1*
DDR2	*DIS3*	*DNMT3A*	*DOT1L*	*EED*	*EGFR*	*EP300*	*EPHA3*	*EPHB1*	*EPHB4*
ERBB2	*ERBB3*	*ERBB4*	*ERCC4*	*ERG*	*ERRFI1*	*ESR1*	*EZH2*	*FAM46C*	*FANCA*
FANCC	*FANCG*	*FANCL*	*FAS*	*FBXW7*	*FGF10*	*FGF12*	*FGF14*	*FGF19*	*FGF23*
FGF3	*FGF4*	*FGF6*	*FGFR1*	*FGFR2*	*FGFR3*	*FGFR4*	*FH*	*FLCN*	*FLT1*
FLT3	*FOXL2*	*FUBP1*	*GABRA6*	*GATA3*	*GATA4*	*GATA6*	*GID4 (C17orf39)*	*GNA11*	*GNA13*
GNAQ	*GNAS*	*GRM3*	*GSK3B*	*H3F3A*	*HDAC1*	*HGF*	*HNF1A*	*HRAS*	*HSD3B1*
ID3	*IDH1*	*IDH2*	*IGF1R*	*IKBKE*	*IKZF1*	*INPP4B*	*IRF2*	*IRF4*	*IRS2*
JAK1	*JAK2*	*JAK3*	*JUN*	*KDM5A*	*KDM5C*	*KDM6A*	*KDR*	*KEAP1*	*KEL*
KIT	*KLHL6*	*KMT2A (MLL)*	*KMT2D (MLL2)*	*KRAS*	*LTK*	*LYN*	*MAF*	*MAP2K1*	*MAP2K2*
MAP2K4	*MAP3K1*	*MAP3K13*	*MAPK1*	*MCL1*	*MDM2*	*MDM4*	*MED12*	*MEF2B*	*MEN1*
MERTK	*MET*	*MITF*	*MKNK1*	*MLH1*	*MPL*	*MRE11A*	*MSH2*	*MSH3*	*MSH6*
MST1R	*MTAP*	*MTOR*	*MUTYH*	*MYC*	*MYCL*	*MYCN*	*MYD88*	*NBN*	*NF1*
NF2	*NFE2L2*	*NFKBIA*	*NKX2-1*	*NOTCH1*	*NOTCH2*	*NOTCH3*	*NPM1*	*NRAS*	*NT5C2*
NTRK1	*NTRK2*	*NTRK3*	*P2RY8*	*PALB2*	*PARK2*	*PARP1*	*PARP2*	*PARP3*	*PAX5*
PBRM1	*PDCD1*	*PDCD1L G2*	*PDGFRA*	*PDGFRB*	*PDK1*	*PIK3C2B*	*PIK3C2G*	*PIK3CA*	*PIK3CB*
PIK3R1	*PIM1*	*PMS2*	*POLD1*	*POLE*	*PPARG*	*PPP2R1A*	*PPP2R2A*	*PRDM1*	*PRKAR1A*
PRKCI	*PTCH1*	*PTEN*	*PTPN11*	*PTPRO*	*QKI*	*RAC1*	*RAD21*	*RAD51*	*RAD51B*
RAD51C	*RAD51D*	*RAD52*	*RAD54L*	*RAF1*	*RARA*	*RB1*	*RBM10*	*REL*	*RET*
RICTOR	*RNF43*	*ROS1*	*RPTOR*	*SDHA*	*SDHB*	*SDHC*	*SDHD*	*SETD2*	*SF3B1*
SGK1	*SMAD2*	*SMAD4*	*SMARC A4*	*SMARC B1*	*SMO*	*SNCAIP*	*SOCS1*	*SOX2*	*SOX9*
SPEN	*SPOP*	*SRC*	*STAG2*	*STAT3*	*STK11*	*SUFU*	*SYK*	*TBX3*	*TEK*
TET2	*TGFBR2*	*TIPARP*	*TNFAIP3*	*TNFRSF14*	*TP53*	*TSC1*	*TSC2*	*TYRO3*	*U2AF1*
VEGFA	*VHL*	*WHSC1*	*WHSC1L1*	*WT1*	*XPO1*	*XRCC2*	*ZNF217*	*ZNF703*	

B. Gene list: select rearrangements of the indicated genes were examined.

ALK intron 18, 19	*BCL2 3 ′ UTR*	*BCR intron 8, 13, 14*	*BRAF intron 7–10*	*BRCA1 intron 2, 7, 8, 12, 16, 19, 20*
BRCA2 intron 2	*CD74 intron 6–8*	*EGFR intron 7, 15, 24–27*	*ETV4 intron 5, 6*	*ETV5 intron 6, 7*
ETV6 intron 5, 6	*EWSR1 intron 7–13*	*EZR intron 9–11*	*FGFR1 intron 1, 5, 17*	*FGFR2 intron 1, 17*
FGFR3 intron 17	*KIT intron 16*	*KMT2A(MLL) intron 6–11*	*MSH2 intron 5*	*MYB intron 14*
MYC intron 1	*NOTCH2 intron 26*	*NTRK1 intron 8–10*	*NTRK2 intron 12*	*NUTM1 intron 1*
PDGFRA intron 7, 9, 11	*RAF1 intron 4-8*	*RARA intron 2*	*RET intron 7–11*	*ROS1 intron 31-35*
RSPO2 intron 1	*SDC4 intron 2*	*SLC34A2 intron 4*	*TERC non-cording RNA*	*TERT promoter*
TMPRSS2 intron 1–3				

Supplementary Table S2. Details of MSI-high cases and MMR gene-mutant cases

MSI-high case

Sample	Primary site	MMR gene	TMB (muts/Mb)	Other gene
SUR-758248	colorectal	Wild	24	ATM mutant (compound hetero)
SUR-758302	esophageal	Wild	19	BRCA2 truncation
SUR-758323	breast	MSH2	82	
SUR-758613	gastric	MSH3	26	

MMR: mismatch repair, TMB: tumor mutational burden

MMR gene-mutant case

Sample	Primary site	MMR gene	Alteration
SUR-758074	breast	*PMS2*	R315*
SUR-758323	breast	*MSH2*	Q409fs*7
SUR-758248	colorectal	*MSH3*	K383fs*32
SUR-758613	gastric	*MSH3*	K383fs*20
SUR-758613	gastric	*MSH3*	K383fs*32
SUR-758831	esophageal	*MSH2*	S612*
SUR-758072	other	*MSH3*	L290fs*10

Supplementary Table S3. The gene list of MMR genes and HRR genes

MMR genes

MLH1	*MSH2*	*MSH6*	*PMS2*	*MSH3*

HRR genes

ARID1A	*ATM*	*ATR*	*ATRX*	*BAP1*	*BARD1*	*BLM*	*BRCA1*	*BRCA2*	*BRIP1*
CDK12	*CHEK1*	*CHEK2*	*FANCA*	*FANCC*	*FANCD2*	*FANCE*	*FANCF*	*FANCG*	*FANCL*
MRE11A	*NBN*	*PALB2*	*RAD50*	*RAD51*	*RAD51B*	*RAD51C*	*RAD51D*	*WRN*	
